# Sub-surface shear wave velocity models developed based on a combined in-situ measurement of quasi-static cone penetration test (q-CPT) and microtremor datasets

**DOI:** 10.1016/j.dib.2024.110501

**Published:** 2024-05-04

**Authors:** Bambang Setiawan, Juellyan Juellyan, Nafisah Al-huda, Alfiansyah Yulianur, Taufiq Saidi, Mark B. Jaksa

**Affiliations:** a Program Study of Geological Engineering, Faculty of Engineering, Universitas Syiah Kuala, Darussalam, Banda Aceh 23111, Indonesia; b Doctoral Student in Engineering, Universitas Syiah Kuala, Darussalam, Banda Aceh 23111, Indonesia; c Department of Civil Engineering, Faculty of Engineering, Universitas Syiah Kuala, Darussalam, Banda Aceh 23111, Indonesia; d School of Architecture and Civil Engineering, University of Adelaide, Adelaide 5005, Australia

**Keywords:** Soil investigation, In-situ testing, Shear wave velocity, Seismic microzonation, Site characterization

## Abstract

A representative sub-surface shear wave velocity model is crucial for seismic hazard studies, as seismic waves are affected by sub-surface characteristics. The offered data in this article were mainly developed based on a quasi-static cone penetration test (q-CPT) collected at the west coast town of Aceh, Indonesia. Microtremor datasets measured at the same locations were employed to extend the depth of the sub-surface models and to validate the models. The in-situ q-CPT data were collected using a locally manufactured Begemann's type cone penetration test apparatus. Twenty seven (27) q-CPT soundings were performed to typical depths of 20 m or measuring cone tip resistances of at least 150 kg/cm^2^. Several empirical approaches were employed to deduce the sub-surface parameters, including shear wave velocity. To enhance the sub-surface model depth, 23 in-situ microtremor data were recorded using 3 components (3C) of Geobit S100 and RaspberrySHAKE (RS-3D) seismometers at the same locations where the q-CPTs were sounded. At the same time, these microtremor datasets were also utilized to validate the developed sub-surface shear wave velocity models using the forward modeling method. Therefore, all the proposed sub-surface shear wave models presented in this article have been validated. These sub-surface shear wave velocity models can be used for site characterization, i.e., site response analysis, seismic microzonation, or spatial urban planning.

Specifications Table

**Table:** 

Subject	Earth and Planetary Sciences
Specific subject area	The Earth and Planetary Sciences field encompasses a broad range of science disciplines including geology and geophysics. This subject also covers applications of geophysics to natural hazards and environmental endeavors.
Data format	Raw (*.mseed), Analyzed (*.pdf)
Type of data	Table, Image, Chart, Graph, Figure
Data collection	In the case of the q-CPT data, during the fieldwork, two direct parameters were measured using manometers: soil resistance to the penetrometer tip; and total soil resistance, being the summation of the penetrometer tip and penetrometer sleeve. By using several empirical approaches these data were used to deduce the soil type, soil unit weight, and soil shear wave velocity. In the case of the microtremor data, the recorded data were converted into the most common wave data format (*.miniSEED) using the Geopsy software package. The horizontal-vertical spectra ratio (HVSR) method was applied to these microtremor data for subsequent analysis. The sub-surface shear wave velocity models were developed by integration of the sub-surface profile based on the q-CPT data into the inversion of the HVSR curve deduced from the microtremor data, as mentioned above. The developed sub-surface shear wave velocity model was validated using the forward modeling method to check the appropriateness of the model.
Data source location	*City/Town/Region: Meulaboh* *Country: Indonesia* *Latitude and longitude (and GPS coordinates) for collected samples/data: [see Figure 1 and Appendix A of the Mendeley Data]*
Data accessibility	With the article and in the following repository data. Repository name: Mendeley Data Data identification number: 10.17632/vrtgv9wyc6.3 Direct URL to data: -https://data.mendeley.com/datasets/vrtgv9wyc6/3 Instructions for accessing these data: -

## 1 Value of the Data

• The q-CPT datasets can be enhanced and compared to other in-situ measurement tools or methods to provide a greater understanding of the repeatability of this method.
• The microtremor datasets can be utilized by seismic hazard researchers for further experiments and studies.
• The proposed sub-surface model is valuable to represent the dynamic characteristics in site response analysis.
• The sub-surface model can be used to provide initial site geotechnical information of the investigated site.
• All the data provided in the present article may be enhanced by various researchers to develop state of the art empirical approaches using machine learning techniques.

## 2 Background

Due to the heterogeneity and the presence of anomalous conditions on the ground, a huge amount of data, i.e., geology, geotechnical, geophysics, and other related data, must be collected and analyzed to deduce the best typical sub-surface characteristics. However, obtaining a large amount of data for the sub-surface characterization can be costly which typically requires drilling boreholes. The need for efficient and economical site characterization in later studies is the main motivation for the development of these datasets. A quasi-static cone penetration test (q-CPT) geotechnical survey and near-surface geophysics of passive seismic survey were carried out for the present paper. A quasi-static cone penetration test (q-CPT) is the most common geotechnical survey in Indonesia. A locally manufactured q-CPT apparatus was employed in this data collection. Near-surface geophysics is a geophysics method that focuses on investigating the sub-surface geological and geotechnical engineering features. In this paper, we provide microtremor datasets recorded using a seismometer. In case an original research article related to the datasets in the present paper, the datasets are used for site response analysis and liquefaction assessment in the city of Meulaboh-Indonesia. The city experienced severe damage due to liquefaction during the M 9.0 December 26, 2004, Sumatra-Andaman earthquake*.*

## 3 Data Description

**Fig. 1:**
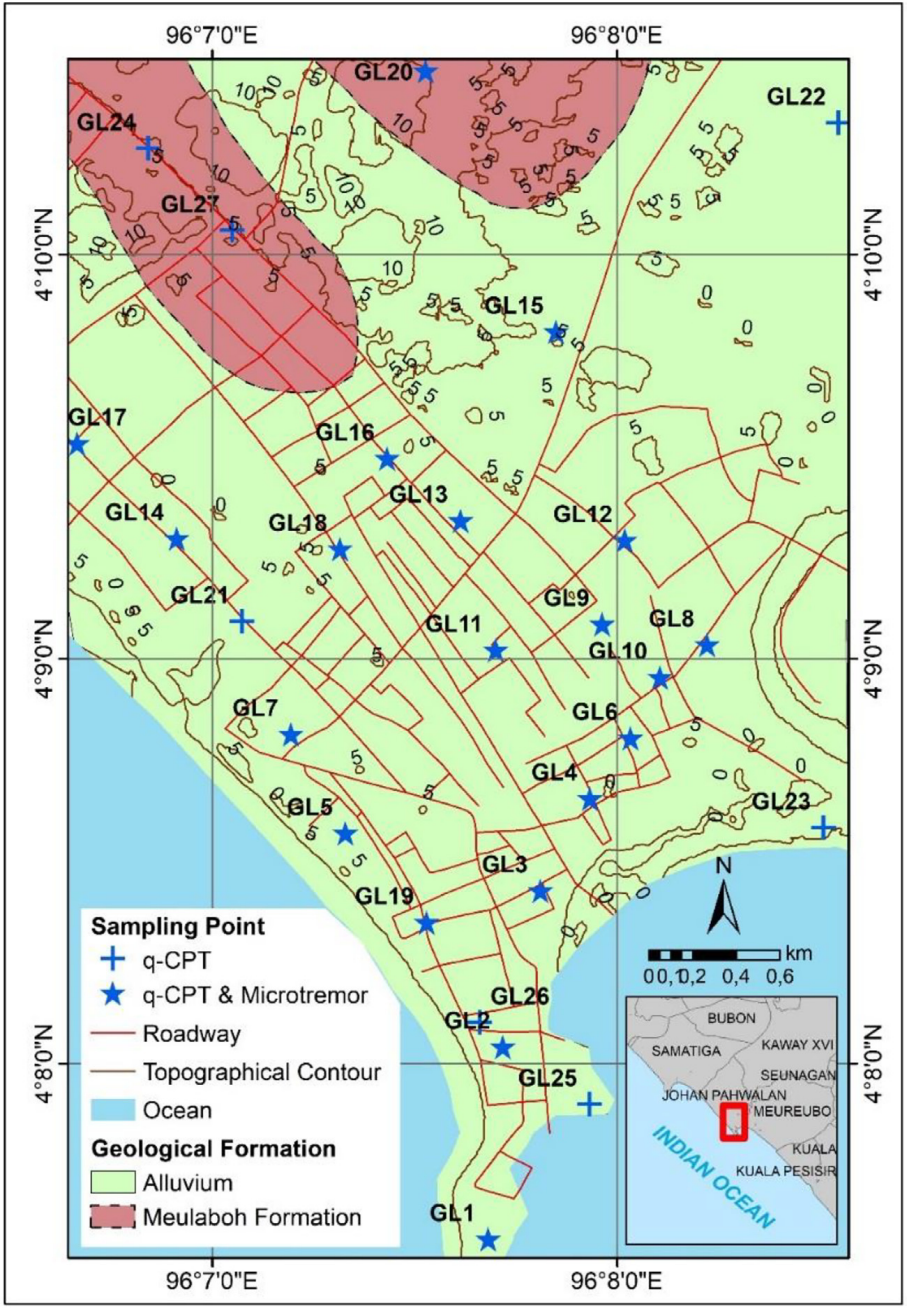
The plotted locations of the recorded q-CPT and microtremor data were incorporated with a geological map of the city of Meulaboh.

The data were collected in the city of Meulaboh, Indonesia. This coastal city is located on the west coast of Aceh, approximately 250 km south-west of the capital city of Aceh province, Banda Aceh (Fig. 1). In the last decade, Meulaboh has been rapidly growing and has become one of the largest urban areas in Aceh province. Since many years ago the city has been an important commercial hub on the west coast of Aceh. The city suffered severe damaged during 2004 Sumatra-Andaman earthquake. Meulaboh and the surrounding areas are characterized by a tropical climate, known as monsoonal, with a wet season from September to May and a dry season between June and August. The precipitations in the collected data area are generally very high in the wet season, averaging 1600 mm/year. The average annual temperature of Meulaboh varies from 24 °C to 35 °C. The city of Meulaboh is within an embayment, called the Meulaboh Embayment, with an altitude between 0 m and 6 m above mean sea level. Geologically, the location of the data collection is composed of the Meulaboh formation (transported cobbles, and sand, clay of Pleistocene age) and alluvium (clay, sand, and gravel of Holocene age) deposits [1].

Cone penetration testing (CPT), which has been practiced since about 1917, basically involves pushing a steel cone attached to rods into the sub-surface to measure the resistance of the soil layer [2]. Further advancement has been made by various researchers, from which the electric cone penetrometer was introduced in Berlin during the Second World War [3]. Many improvements of the electric CPT relative to the mechanical cone penetrometer, namely the ability of the equipment to eliminate errors caused by friction between the inner and outer rods; longer continuous testing up to 1-m length with a steady rate of penetration that minimizes undesirable soil movement; and more reliable measurements have been made [2]. However, the use of mechanical cone penetrometers in soil investigation is still very common in Indonesia.

The mechanical cone penetration test, known as the Sondir in Indonesia, procedure is standardized by ASTM D-3441-75T [3] and SNI 2927:2008 [4]. This test also is called the mechanical cone penetration test or quasi-static cone penetration test (q-CPT). In the present article, we use this latter term. In geotechnical engineering, the q-CPT data are commonly used for several purposes such as a) sub-surface stratigraphic/soil profiling evaluation, soil layer classification, soil strength, and bedrock depth; b) determining the thickness of overburden sediment; c) footing design including settlement calculation; and d) liquefaction assessment.

**Fig. 2:**
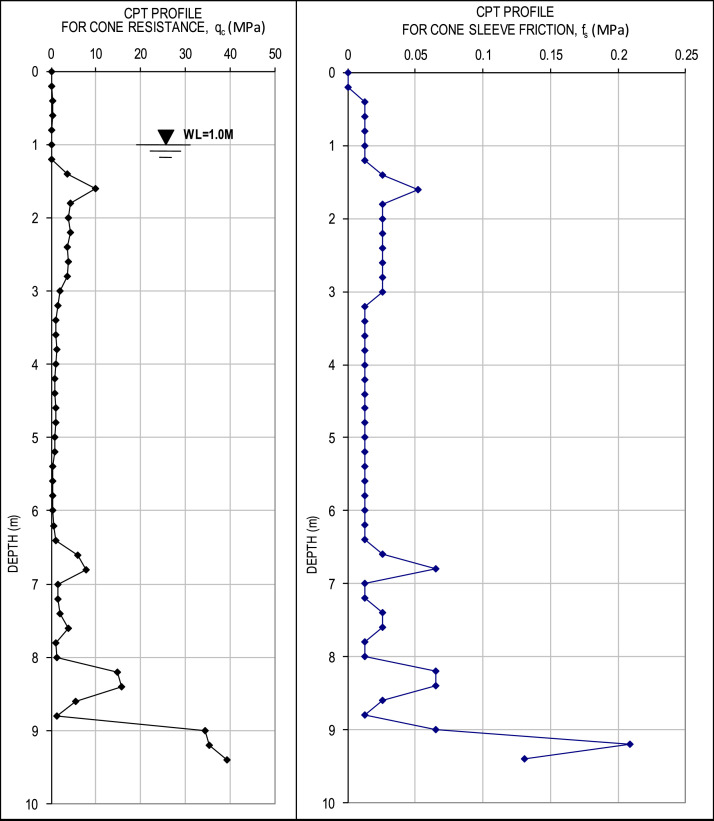
Example of the q-CPT data.

The raw q-CPT data consist of two recorded parameters: cone tip resistance and the sleeve friction, with both being in units of kg/cm² and measured at depth increments of 0.2 m. In this article, we have converted the unit of the q-CPT data into MPa. An example of the data is presented in Fig. 2. All the completed q-CPT data are included in Appendix B of the Mendeley Data.

**Fig. 3:**
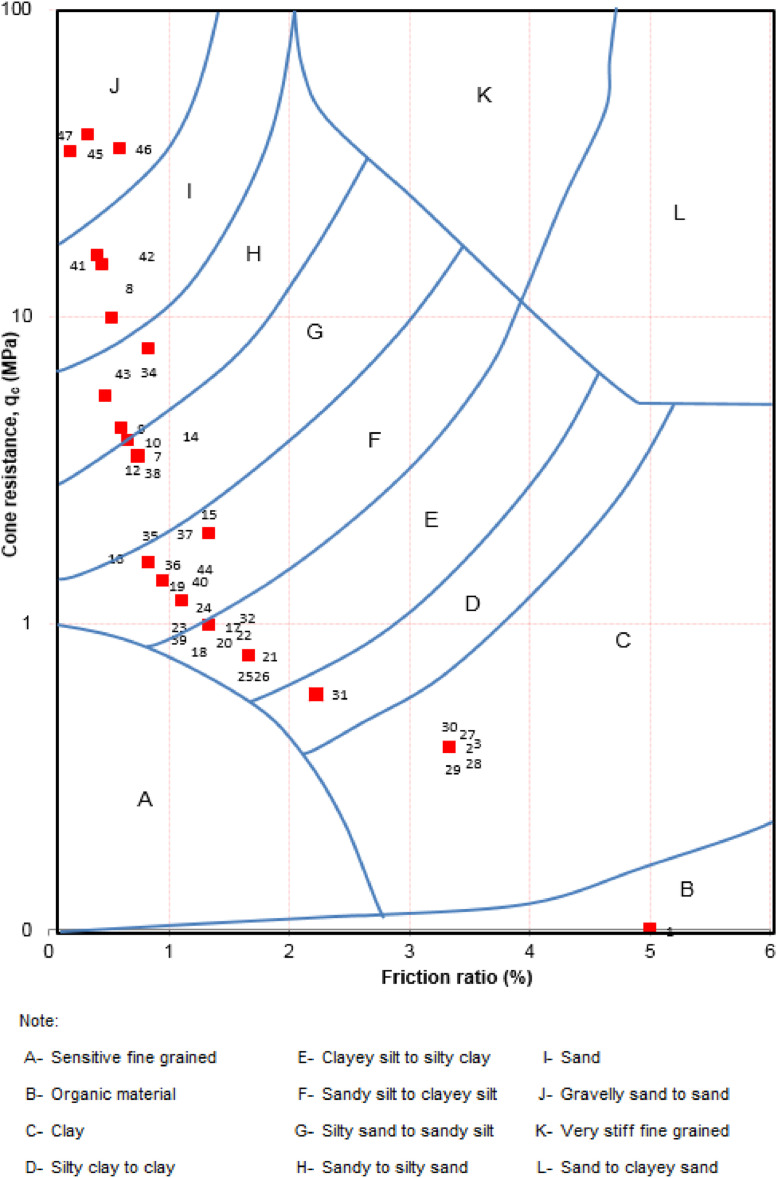
Example of enhancing q-CPT data for soil classification using the empirical approach of [5].

The raw q-CPT data were enhanced to obtain soil type using the empirical approach of [5]. This approach uses cone tip resistance data (q_c_ in a units of MPa) and friction ratio, which is a ratio of the sleeve friction divided by cone tip resistance, expressed as a percentage. Ref. [5] classifies 12 soil types based on these two q-CPT parameters, as shown in Fig. 3. All the enhancing q-CPT data for soil classification using the empirical approach is included in Appendix C of the Mendeley Data.

**Fig. 4:**
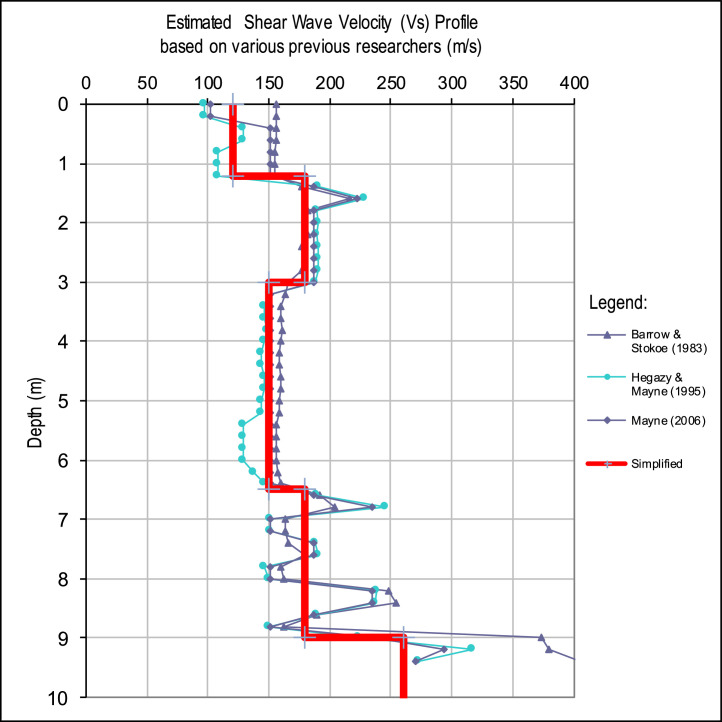
Example of the generated shear wave velocities based on the q-CPT data.

Another important variable that can be estimated from the q-CPT data is soil shear wave velocity. Several empirical approaches [6], [7], [8], were employed in this process. An example of generated shear wave velocity profile based on the q-CPT empirical approaches is presented in Fig. 4. A simplified shear wave velocity profile was proposed from the generated shear wave velocity profile based on the q-CPT data, as shown by the red line in Fig. 4. All of the enhanced q-CPT data used to estimate the shear wave velocity from the empirical approaches are included in Appendix D of the Mendeley Data.

**Fig. 5:**
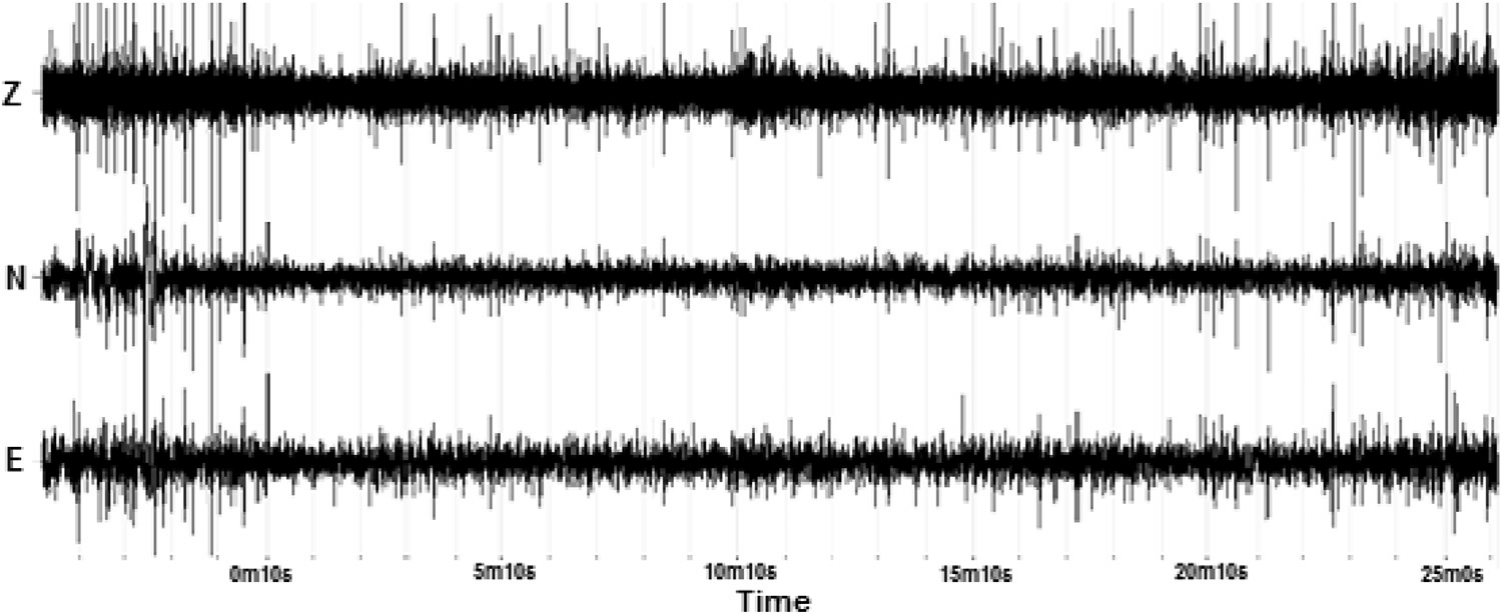
Example of the raw microtremor data.

The raw microtremor data were recorded by setting a triaxial sensor seismometer on a generally firm to stiff ground surface at the targeted location. In the present paper, at least one-hour of microtremor data are considered enough to represent the measured location. The raw recorded data were converted into the most common wave data format (*.miniSEED) using the Geopsy software package [9]. An example of the raw microtremor data is presented in Fig. 5. Three microtremor waveforms of Z, N, and E were recorded by the seismometer; where Z refers to the vertical-horizontal direction waveforms, N the north-south direction waveforms, and E the east-west direction waveforms. All the raw microtremor data are included in Mendeley Repository Data (https://data.mendeley.com/datasets/vrtgv9wyc6/3).

**Fig. 6:**
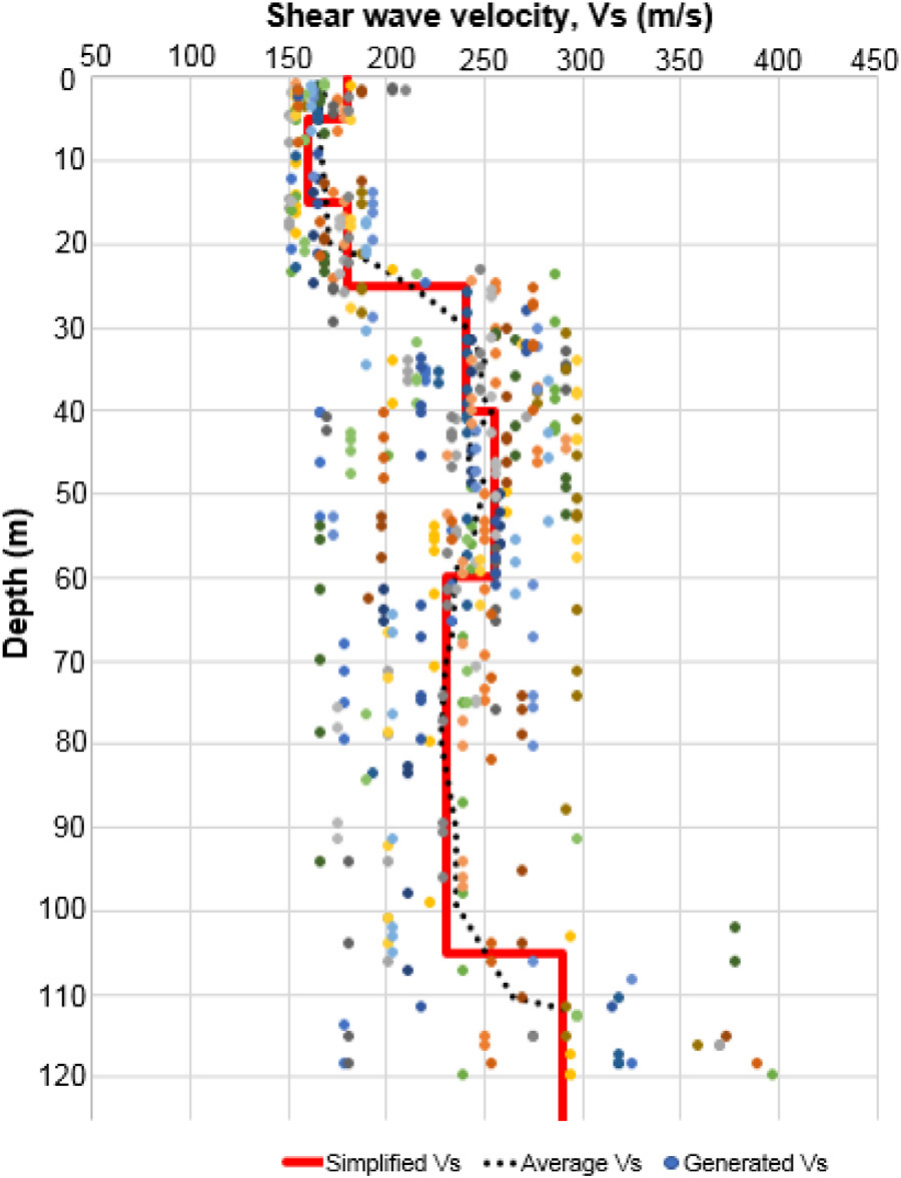
An example of the 20 best-generated shear velocity models (shown in various colors of small dots) including the simplified one.

To generate the initial sub-surface shear wave velocity profile at the measured location (Fig. 1), an inversion of the horizontal-vertical spectra ratio (HVSR) curve was carried out using the Dinver software package within the Geopsy software package [9]. The best 20 generated models were extracted. An example of this generated shear velocity model, including the simplified one, is shown in Fig. 6. All of the generated shear velocity models using this inversion are included in Appendix E of the Mendeley Data.

**Fig. 7:**
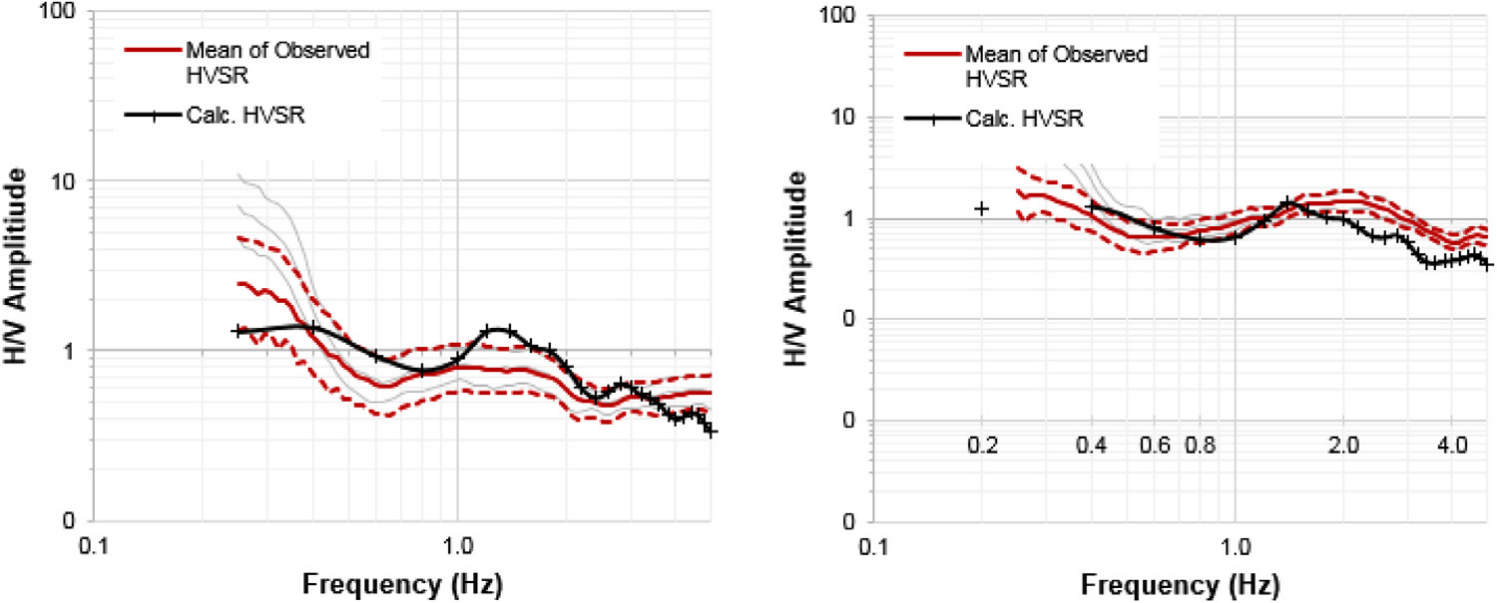
A validation using the forward modeling method.

**Fig. 8:**
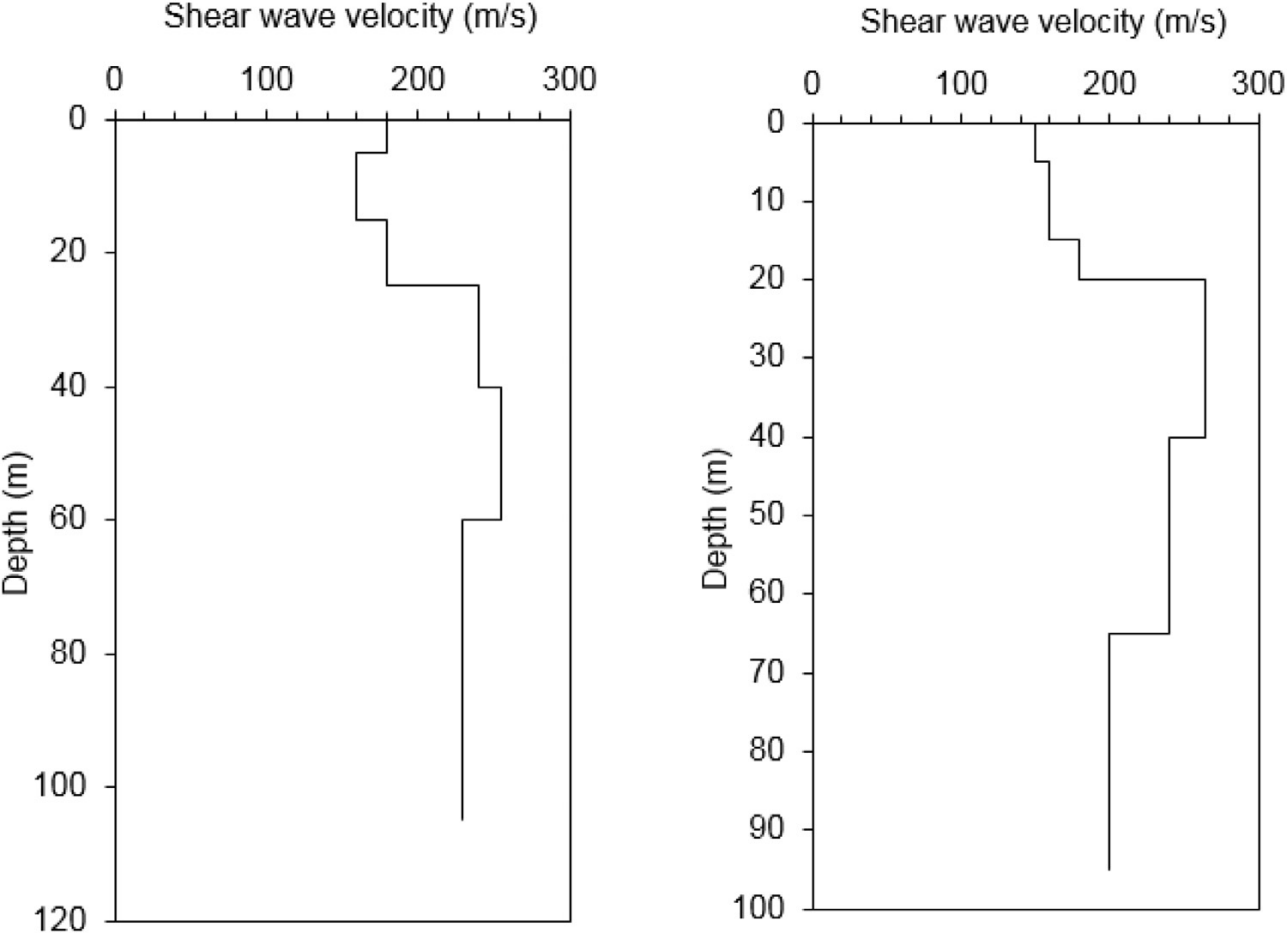
Final sub-surface shear wave velocity models.

The final sub-surface shear wave velocity models were developed by integration of the sub-surface profile based on the q-CPT data into the inversion of the HVSR curve deduced from the microtremor data, as mentioned above. A validation using the forward modeling method was carried out to check the appropriateness of the model. Two of the validation data are presented in Fig. 7. All of the validation data using the forward modeling method is included in Appendix F of the Mendeley Data. As aforementioned, the present paper focuses on the development of sub-surface shear wave velocity models in the city of Meulaboh, Indonesia (Fig. 1). One of the final sub-surface shear wave velocity models is presented in Fig. 8. All of the proposed shear wave velocity models are given in Appendix G of the Mendeley Data.

## 4 Experimental Design, Materials and Methods

### 4.1 Quasi-static cone penetration test (q-CPT)

The q-CPT is an invasive soil test that currently is widely used for soil investigation in Indonesia. This in-situ testing is highly repeatable. The test consists of pushing a cone of standard dimensions vertically into the ground at a constant rate while recording its resistance at regular depth intervals. The basic data measured by a standard CPT are the tip resistance, q_c_, sleeve friction, f_s_, and the respective depth of the penetrometer.

### 4.2 Equipment

**Fig. 9:**
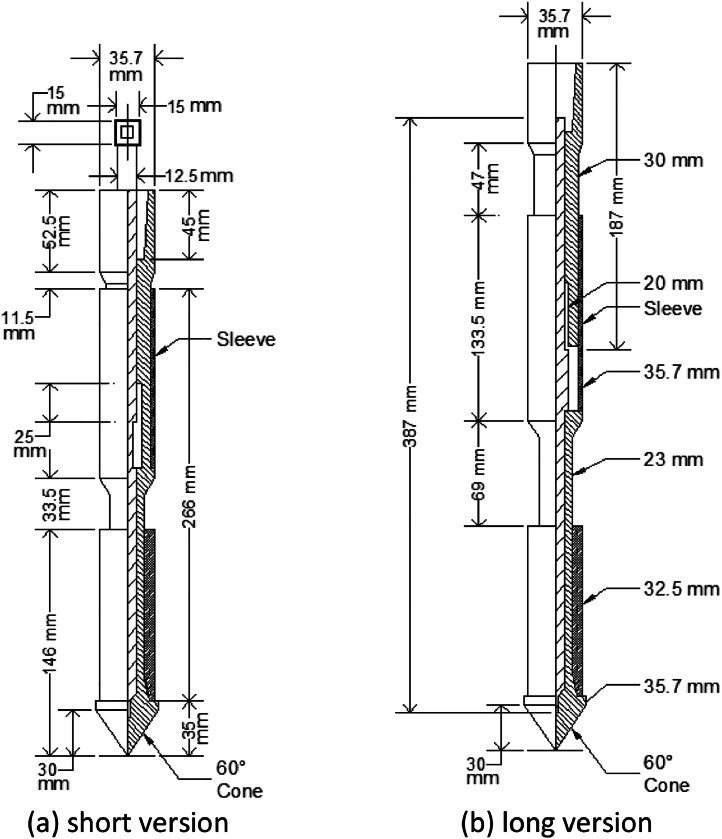
Biconal penetrometer or Begemann penetrometer type.

**Fig. 10:**
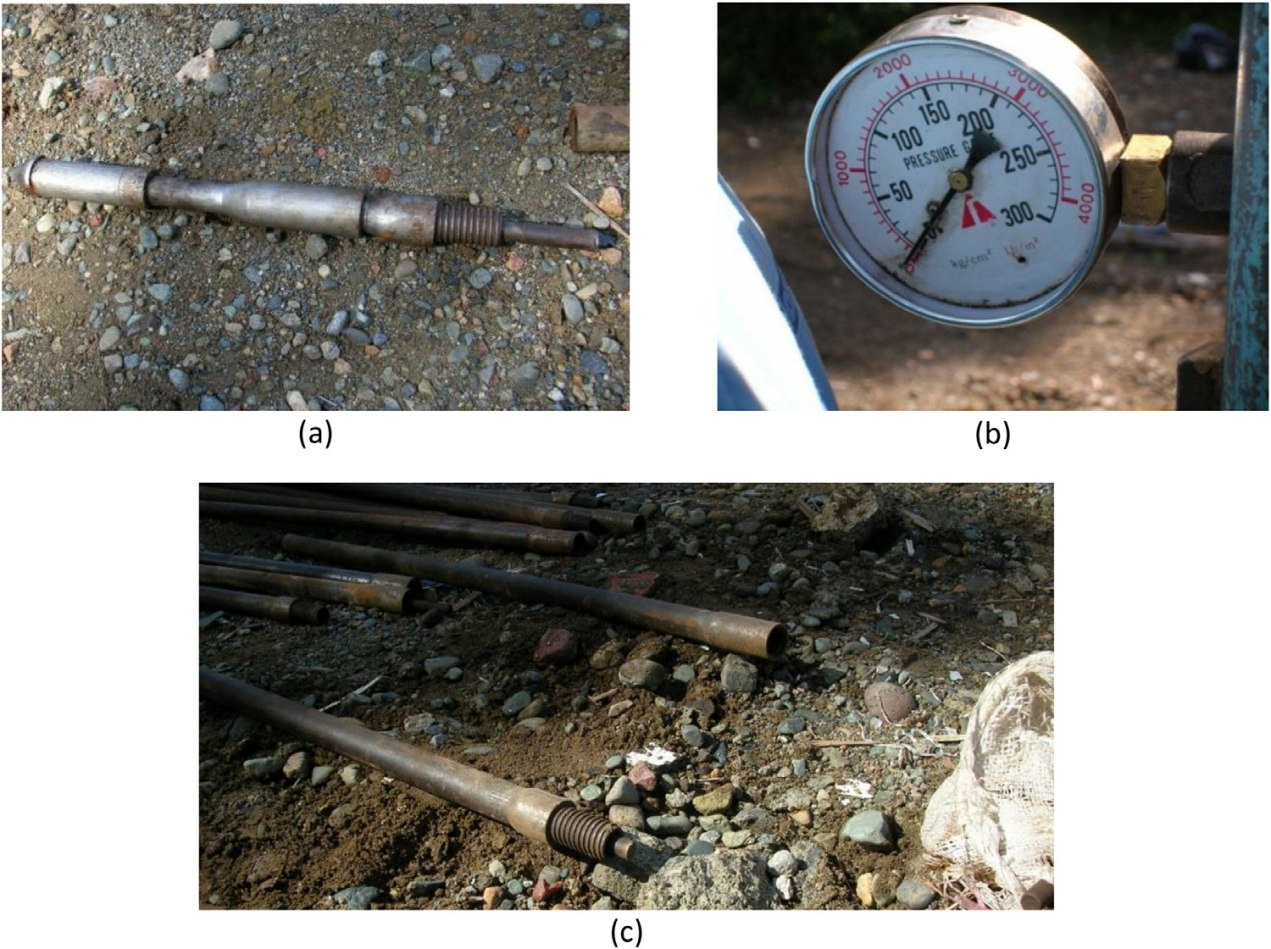
q-CPT equipment: (a) cone penetrometer, (b) manometer, and (c) rods.

The adopted q-CPT equipment used to acquire the data is a kind of Dutch Cone penetrometer with a capacity of two tons. The used penetrometer is working mechanically. This penetrometer has a conical tip and sleeve (biconal) or Begemann penetrometer type (Figs. 9 and 10a). A cone of standard dimensions has a diameter of 35.7 mm ± 0.4 mm, a projection of cross-sectional area of 1000 mm^2^ with an apex angle of 60° ± 5°, and a friction sleeve with a surface area of 15,000 mm^2^ ±300 mm^2^ and a length of 133.5 mm+up to 0.5 mm, as shown in Fig. 9. This cone must made of steel (Fig. 10a).

**Fig. 11:**
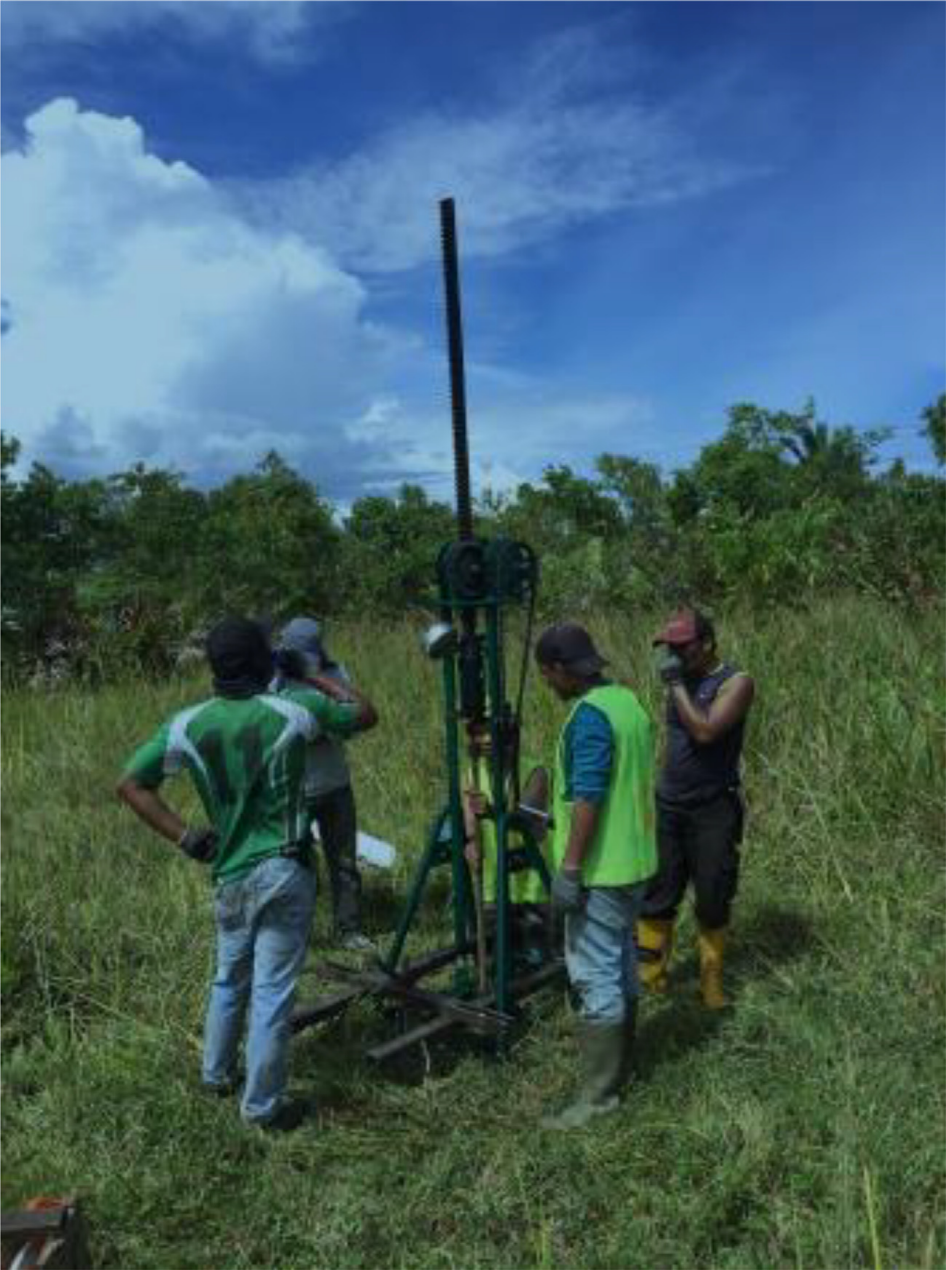
Field application of q-CPT equipment.

The cone penetrometer is connected to the grip or adapter of the pushing system by cone rods designed to reach the testing depth. A cone-rod is typically 35.7 mm in outer diameter and manufactured from hollow steel, as shown in Fig. 10b. Usually, the rods are one-meter in length with tapered threads. A stack of 20–40 one-meter long rods is usually necessary for most geotechnical site investigations. A manometer, as shown in Fig. 10c, is used to measure the resistance of the soil. The whole assembly of the q-CPT equipment in the field is shown in Fig. 11.

### 4.3 Procedure

The general CPT procedure is standardized by various organizations [10], such as the American Society for Testing and Materials (ASTM-D3441-75T-1987) [4], Standard National Indonesia (SNI 2827:2008) [4], the International Society of Soil Mechanics and Foundation Engineering (ISSMFE-1989) [11], Standards Australia (AS 1289.6.5.1-1999) [12]. The detailed procedure of the q-CPT adopted in the present paper is outlined by SNI 2827:2008 [4].

In certain cases, such as testing an area with a topsoil that consists of fill or hard soils, it may be necessary to pre-drill in order to avoid damaging the cone equipment. This may be carried out by using a solid dummy probe with a diameter slightly larger than the cone penetrometer or manually drilling an adequate hole to the required depth.

The thrust machine must be set up for a thrust direction as near to vertical as is practicable, with a maximum deviation of the push rods of only 2°. This precision is aimed at avoiding damage to the equipment due to a sudden deflection and ensuring that the penetrometer remains vertical. However, deflection caused by the impact of hard strata or inclusions is not uncommon. Generally, 1° deflection per meter without noticeable damage is acceptable.

The speed of the penetration into the ground should be set and maintained at between 1 and 2 cm/s. A manometer dial reading is taken at every 20 cm penetration. Two manometers working in unison are used to measure the pressure (q_c_ and f_s_) exerted by the penetration.

### 4.4 Applications and data interpretation

Although q-CPT in-situ testing retrieves no sample for laboratory testing or visual inspection, this in-situ test has the capacity to produce several soil parameters [13]. The information obtained from the q-CPT can be used to interpret the following soil characteristics: classification of soils, relative density of granular soils, friction angle of granular soils, undrained shear strength of cohesive soils, overconsolidation ratio of soils, Young's modulus of elasticity, compressibility of clay, bearing capacity of deep and shallow footing, and liquefaction potential of soils.

### 4.5 Microtremor measurement method

The microtremor measurement method has been found to be useful in investigating the near-surface geology. This near-surface investigation is mainly concerned with seismic hazard assessment (see [14], [15], [16], [17], [18], [19]).

### 4.6 Equipment

**Fig. 12:**
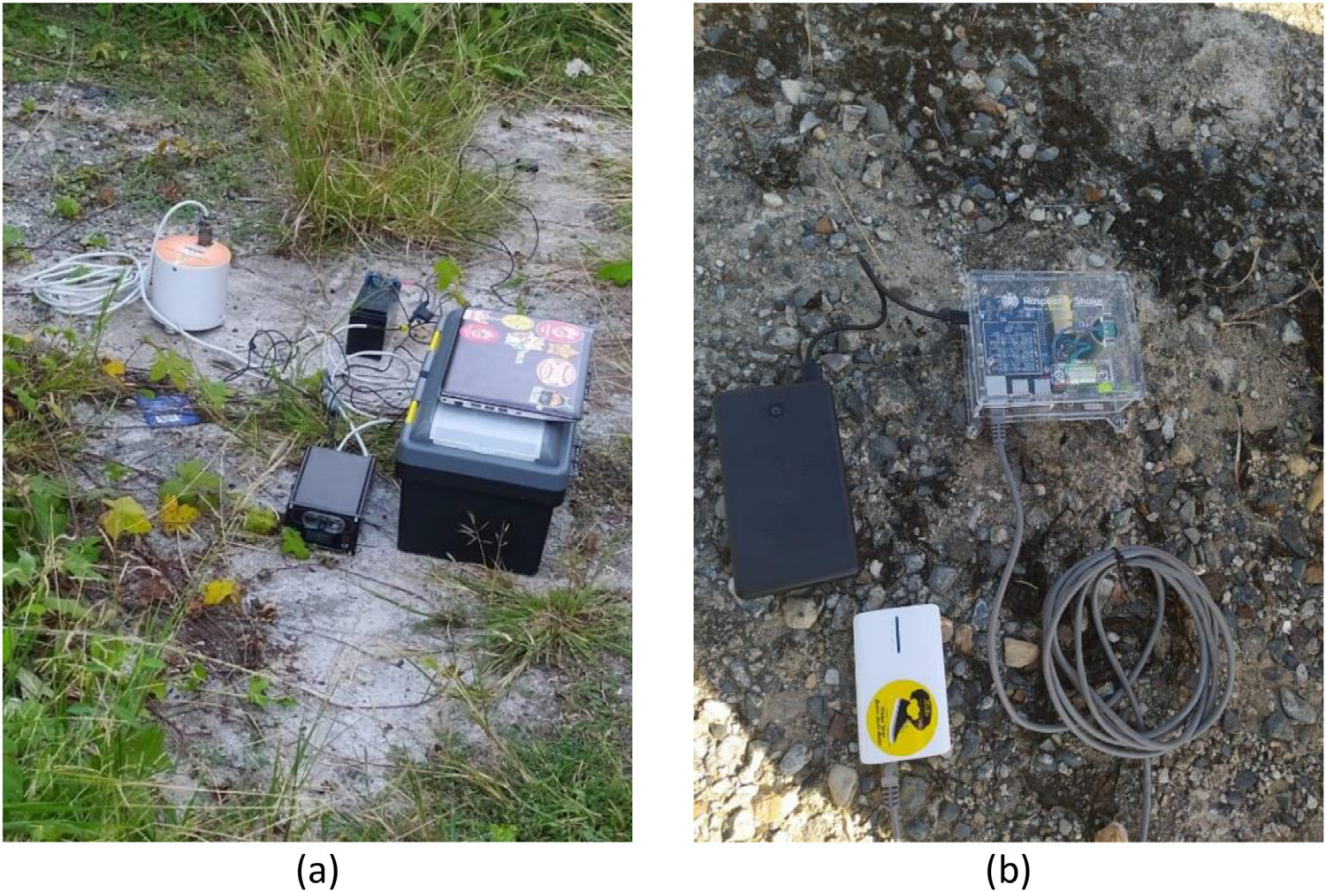
Seismometers adopted in the present paper: (a) Geobit S100 Wide Band seismometer, and (b) Raspberry Shake 3D (RS-3D) seismometer.

A seismometer is mainly used in the microtremor measurement method. In the present paper, two sets of seismometers (Fig. 12) were employed to record the data. The first one is the Geobit S100 Wide Band seismometer, as shown in Fig. 12a. This surface-type Geobit S100 seismometer (ST Geobit S100) is an ultra-lightweight (1100 g) seismometer with a three-axis velocity sensor in a sealed IP67 case. This seismometer can measure microtremor vibrations over a wide frequency range of 0.1 to 98 Hertz simultaneously. The ST Geobit S100 incorporates a data logger which saves the data to a micro SD card.

The second is a low-cost Raspberry Shake 3D (RS-3D) seismometer, as shown in Fig. 12b. This RS-3D integrates 3 orthogonal velocity sensors, the digitizers, the hyper-dampers, and the microcomputer into a single box. The adopted sensor in the RS-3D can be electronically extended down to 0.5 Hz. The recorded data are saved into an 8 GB internal storage with an additional micro SD card.

Both the ST Geobit S100 and RS-3D seismometers use cable connectors and a battery power supply for field measurement. An additional GPS antenna can be used in these seismometers for accurate timing (if required).

### 4.7 Procedure

**Fig. 13:**
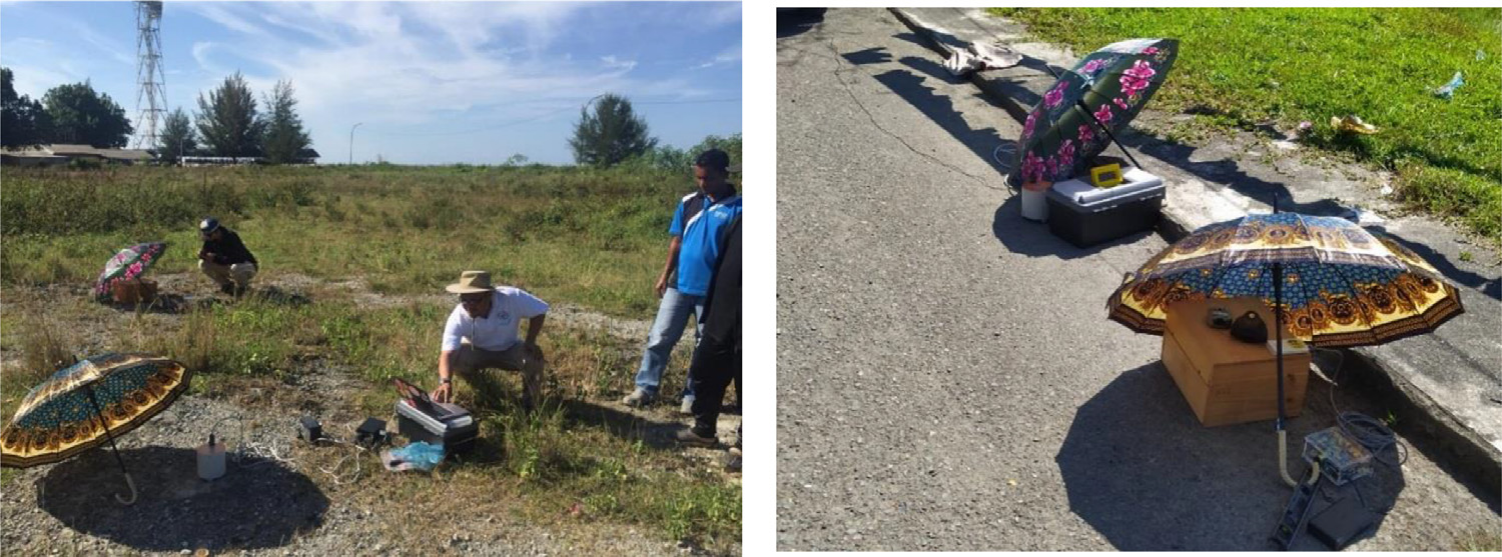
Field microtremor measurement.

For the target location of the seismometer, prior to recording, generally firm to stiff ground was selected and cleared of obstacles, such as roots, pebbles, and tall grass. If required, the ground may be leveled so that the seismometer is stable during data recording. Three legs on the seismometer can be used for modest level adjustment of the device. The seismometer was then oriented to north and protected from the sun and rain by means of an umbrella. A portable computer was employed for initial setup and checking. A small portable battery was used to generate the system power. In the present paper, at least one-hour of microtremor data is considered enough to represent the measured location. The field microtremor measurement is shown in Fig. 13, and the detailed procedure is outlined in [20].

### 4.8 Applications and data interpretation

Microtremor data have been applied to deduce the near-surface geology characteristics, which is mainly in seismic hazard assessment, including seismic microzonation, site amplification, and soil liquefaction. Microtremor data can be applied also for general geotechnical investigations, such as slope stability assessment, road pavement quality control, and fill compaction evaluation. Microtremor datasets can also be used to interpret various sub-surface parameters, such as site predominant frequency, soil overburden thickness, soil shear wave velocity, and sub-surface geometry.

## Limitations

There are several limitations related to the datasets presented in this paper, as follow:

­ There are no samples provided in the datasets in the present paper.
­ The q-CPT datasets were collected to the limited depth.
­ The microtremor datasets are recorded in the limited durations.
­ Currently, enhancement of microtremor datasets for deducing other sub-surface parameters is rather limited when compared to q-CPT data. Furthermore, there is no approach currently available to estimate soil types using microtremor datasets.

## Ethics Statement

The authors have read and follow the ethical requirements for publication in Data in Brief and confirming that the current work does not involve human subjects, animal experiments, or any data collected from social media platforms.

## CRediT authorship contribution statement

**Bambang Setiawan:** Conceptualization, Methodology, Software, Data curation, Writing – original draft, Software, Visualization, Validation, Writing – review & editing. **Juellyan Juellyan:** Conceptualization, Methodology, Software, Data curation, Writing – original draft, Software, Visualization, Validation. **Nafisah Al-huda:** Conceptualization, Methodology, Software, Software, Visualization, Validation. **Alfiansyah Yulianur:** Investigation, Supervision. **Taufiq Saidi:** Investigation, Supervision. **Mark B. Jaksa:** Writing – review & editing.

## Data Availability

Raw waveform microtremor data recorded in the City of Meulaboh (Original data) (Mendeley Data).
